# The Effect of Occupational Health Risk Perception on Job Satisfaction

**DOI:** 10.3390/ijerph19042111

**Published:** 2022-02-13

**Authors:** Biaoan Shan, Xiaoju Liu, Anwei Gu, Runxuan Zhao

**Affiliations:** School of Business and Management, Jilin University, Changchun 130022, China; shanbiaoan@jlu.edu.cn (B.S.); 15344052394@163.com (X.L.); zrxqweaz@163.com (R.Z.)

**Keywords:** occupational health risk perception, job satisfaction, work stress, organizational commitment, safety culture

## Abstract

This study explored the relationship between occupational health risk perception and job satisfaction. Based on the job demand-resources model and resource conservation theory, eight hypotheses were proposed in this study. In a survey of 237 production line workers and managers, we found that perceived occupational health risks significantly negatively affected job satisfaction. Both work stress and organizational commitment mediate the relationships between perceived occupational health risks and job satisfaction. We also examined whether safety culture could weaken the negative impact of perceived occupational health risks on job satisfaction. However, the results of our study did not support this hypothesis. This study not only helped managers to realize the hazards of occupational health risks, but also encouraged employees to actively participate in safety construction and pay attention to their own health. In addition, we also put forward some targeted intervention measures to reduce the negative impact of perceived occupational health risks on job satisfaction. Therefore, this study had certain practical implications.

## 1. Introduction

The problem of occupational safety and health has always been a concern of scholars and practitioners. At the beginning, scholars mainly focused on occupational safety problems with a high hazard degree, and that were intuitive and easy to measure. In recent years, more and more scholars have started to pay attention to the topic of occupational health risks faced by employees, which is a long-term, hidden, and easily ignored problem. In order to protect the physical and mental health of workers, many countries have issued a series of policies. In some industries, there are even strict rules to improve the working environment for employees. The Chinese government also attaches great importance to the occupational health of employees. Various industries have issued a series of occupational health and safety laws and standards (such as the Standard of Environment and Sanitation of Construction Site, and the Occupational Health and Safety Management Systems-Requirements).

Employees are the core assets of an enterprise. The working environment and perceived occupational health risks of employees are crucial to individual work output and the development of the enterprise. This is an important topic with practical significance [1], because the degree of perceived health risks will largely determine employees’ job satisfaction. The higher the workers’ subjective evaluation of their work environment safety (or the less external health risks employees perceive), the lower their perceived work stress [2]. Work stress will negatively affect employees’ job and life satisfaction. The job satisfaction of employees is the key driving force for their value creation, creativity, and job performance in the organization [3], which will greatly affect employees’ working state and decision-making behavior. However, studies have found that perceived occupational health risks can harm employees’ physical and mental health. Perceived occupational health risks may lead to high stress states and low organizational commitment, which negatively affects employees’ job satisfaction [2,4,5,6]. This also means that the presence of perceived occupational health risks may be detrimental to employees’ job satisfaction. 

It is a pity that this critical issue has been overlooked in the existing literature. From existing studies, previous scholars have mainly focused on the concept and measurement of occupational health risk perception, and evaluated which factors affect employees’ occupational health risk perception, and how employees and managers should deal with occupational health risks [7,8,9]. The mechanism analysis of the impact of perceived occupational health risks on job satisfaction has been neglected [10,11]. This means that we are unable to find out the path of the impact of occupational health risk perception on job satisfaction through existing literature, nor can we reduce the negative impact of occupational health risk perception on job satisfaction through interventional measures in enterprise practice. In particular, how do perceived occupational health risks negatively affect job satisfaction? Can organizational situational factors mitigate this negative effect to some extent? An in-depth analysis of these problems will help enterprises in some industries to better understand the adverse effects of occupational health risks perceived by employees and find effective solutions to these problems theoretically.

Based on the above analysis, this study attempts to accept this challenge and explore the influence mechanism of occupational health risk perception on employees’ job satisfaction through a theoretical analysis and questionnaire survey. This study suggests that perceived occupational health risks indirectly affect job satisfaction through psychological states of work stress and organizational commitment. We used work stress and organizational commitment as mediators to reveal the negative impact of perceived occupational health risks on job satisfaction. At the same time, according to the theoretical framework of the job demands-resources model, safety culture, as a resource to protect employees from negative influences of the work environment (such as occupational health risks), will regulate the relationship between risk perception and job satisfaction [12]. When employees perceive the differences in organizational safety culture, safety planning, and other aspects, they may adopt completely different attitudes (positive or negative) to deal with perceived occupational health risks, resulting in different job satisfaction. Therefore, we take organizational safety culture as a situational variable into the research framework in order to further deepen the logical relationship between perceived occupational health risks and job satisfaction.

This study will help enterprise managers have a deeper understanding of the importance of workplace safety environment and will theoretically guide them so as to reduce the negative impact of employees’ occupational health risk perception. The rest of the paper is structured as follows. We first review the literature on occupational health risk perception and job satisfaction. Then, we propose hypotheses based on theories and provide a theoretical framework. After that, research methods and data sources are explained. Fourth, we analyze the data and present the empirical results. Next, we discuss the results, and analyze the theoretical and management implications, and the limitations of the research. Finally, the conclusions of this paper are expounded.

## 2. Literature Review and Hypotheses

### 2.1. Occupational Health Risk Perception

Occupational safety and health have always been the focus of scholars, employees, and entrepreneurs. The harm caused by occupational safety accidents to employees is more intuitive, such as falling from great height, traffic accidents, and so on. Compared with occupational safety accidents, occupational health risks are relatively hidden, and their hazards can only be found after a long time, such as excessive exposure to sunlight, excessive inhalation of formaldehyde, and so on. Occupational health risks refer to employees’ exposure to an environment harmful to the human body due to work reasons, which may lead to employees’ physical or mental health problems [1].

Employee behavior and job performance are determined by many factors, such as environmental factors, behavioral factors, and cognitive factors. Cognitive factors include knowledge, perception, attitude, and expectation [13,14]. Employees’ cognition and judgment of occupational health risks affect their psychological state and work results to a great extent. Sometimes, employees’ subjective perception of the work environment is more important than their objective evaluation [15]. Therefore, compared with the objective evaluation of occupational health risks, employees’ subjective perception of occupational health risks may affect employees’ psychological state and work results to a greater extent.

According to the risk perception model, risk perception includes rational perception and emotional perception [16]. Employees’ rational perception of occupational health risks refers to the judgment of the probability and severity of occupational health hazards. Employees’ emotional perception of occupational health risks refers to their emotional reaction to the occurrence of occupational health hazards. This study holds that employees’ perception of occupational health risks refers to the process of assessing the possible risks at work and forming their perception of job characteristics based on their perception of the environment. This process reflects employees’ awareness and understanding of occupational health risks.

The existing research on occupational health risk perception mainly focuses on the construction field, transportation field, medical industry, pollution treatment industry, and other fields. Existing studies mainly focus on qualitative or quantitative occupational health risk perception, assessing which factors will affect employees’ occupational health risk perception, and what employees and managers should do to deal with occupational health risks. The main factors affecting occupational health risk perception are individual differences (such as age, experience, personal knowledge, and education level), the influence of information, the nature of risk characteristics, and so on [7,17]. Portell et al. (2014) [8] took medical staff as the research object and described nine dimensions of occupational risk perception from an individual level, namely individual knowledge, expert knowledge, fear, weakness, severity, the possibility of avoidable and controllable harm, and immediacy. Liu et al. (2021) [1] considered that the occupational health risk perception of indoor construction workers will significantly affect the risk response behavior of employees, which provided a new research perspective for the occupational health of construction workers. Zhou (2014) [9] took bus drivers as the research object and provided specific suggestions for bus drivers by understanding their views on extreme heat and occupational health risks. Sapkota et al. (2020) [13] also explored how informal waste recycling workers perceive and reduce the risks associated with waste recycling.

### 2.2. Job Satisfaction

Job satisfaction is closely related to employees’ occupational health and is one of the main indicators to measure the quality of employees’ work and life [18]. Job satisfaction is a kind of work result and work output. Job satisfaction largely depends on the working environment and employees’ perception of the working environment [4]. Existing studies mainly focus on the relationship between emotion (positive emotion and negative emotion) and job satisfaction. Scholars believe that personal emotion makes employees differently sensitive to external stimuli. This leads to different reactions and cognition of employees to the same working environment, resulting in different job satisfaction [19]. Hmieleski and Corbett (2008) [20] regarded job satisfaction as an important result of entrepreneurs’ impromptu behavior, and believed that job satisfaction may be a more important success indicator than financial performance. They explored the moderating effect of entrepreneur self-efficacy on the relationship between improvisation, entrepreneurial performance, and individual job satisfaction. Referring to the viewpoint of Hmieleski and Corbett (2008) [20], this study believes that job satisfaction is an important work result and output produced by employees under the comprehensive effect of various conditions, such as the work environment and work demand. Job satisfaction reflects the degree to which a job meets the needs of employees.

### 2.3. Occupational Health Risk Perception and Job Satisfaction

Job satisfaction, as an outcome of employees’ work, is closely related to occupational health risks perceived by employees, and extensively affects organizational behavior and results [21]. When employees feel low job satisfaction, they are unwilling to accept the organization’s goals and values, and may even consider leaving the organization [22]. However, the existing literature lacks research on the impact mechanism between employees’ occupational health risk perception and employees’ job satisfaction. Therefore, it is necessary to study the impact mechanism between employees’ occupational health risk perception and their job satisfaction. 

Job satisfaction is closely related to working conditions and job characteristics. Unsafe and unhealthy work characteristics in the workplace will negatively affect employees’ job satisfaction [4]. According to the theoretical framework of the job demands-resources model, each type of job characteristics is divided into job demands and job resources [23]. Job demands refer to the psychological, physical, social, and organizational requirements of work, which requires individuals to continuously pay physical and psychological efforts and costs. Job resources refer to the physical, psychological, social, or organizational factors that have the function of achieving work goals, reducing work demands, and realizing personal value. Empirical research shows that there is a negative correlation between job demands and job satisfaction. Job demands such as occupational hazards and occupational risks are closely related to the damage of job satisfaction [12].

Occupational health risk will harm employees’ physical or mental health, which is essentially a kind of job demand. According to the theoretical framework of the job demands-resources model, job demands negatively affect job satisfaction. Therefore, occupational health risk perception, as employees’ perception of job demands, will reduce employees’ job satisfaction. Therefore, this study proposes the following hypothesis:

**Hypothesis** **1** **(H1).** *Occupational health risk perception negatively affects job satisfaction.*

### 2.4. The Moderating Effect of Employee Perception of Safety Culture

Organizational safety culture is a comprehensive consideration of safety policies, safety procedures, and safety practices [24]. Safety culture can be measured from eight dimensions, which are managers’ commitment to safety, safety priority, communication, safety rules, support environment, participation, personal risk assessment, and work environment [25]. Safety culture is the leading factor of safety results and can create an environment to reduce employee risks and injuries [2]. The higher the employee’s perceived occupational health risks, the lower their job satisfaction. A positive safety culture can reduce the negative impact of occupational health risk perception on job satisfaction [12].

According to the theoretical framework of the job demands-resources model, there is a positive correlation between job resources and job satisfaction [23]. Safety culture is a form of safety supervision and support, which can be regarded as job resources. As a resource to protect employees from the negative impact of job demands (such as occupational health risks), safety culture can regulate the relationship between risk perception and job satisfaction [12]. When employees perceive a high level of organizational safety culture and good safety planning, employees will feel that their safety and health are concerned by the organization. At this time, employees will more actively deal with their perceived occupational health risks, resulting in a higher job satisfaction. On the contrary, when employees perceive a low level of organizational safety culture and poor safety planning, employees will feel that their safety and health are not a concern of the organization. At this time, employees will be more negative when dealing with their perceived occupational health risks, resulting in lower job satisfaction. Therefore, we argue the following hypothesis:

**Hypothesis** **2** **(H2).** *Safety culture weakens the negative impact of occupational health risk on job satisfaction.*

### 2.5. The Impact of Occupational Health Risk Perception on Work Stress and Organizational Commitment

Work stress refers to the impact of workplace stressors on employees’ psychological and physical health. This effect may be short-term or long-term [26]. Some scholars believe that stress arises from people’s comparison of demands and resources. When demands exceed resources and endanger people’s happiness, stress occurs [27]. This study believes that work stress is a psychological state formed after employees perceive that their job demands are greater than their perceived work resources. An unsupportive work environment is one of the most important determinants of work stress [28]. Work environments that lack safety support can contribute to work stress. According to the transactional model of stress, events in the work environment involve cognitive assessment processes. The primary assessment includes assessing whether the event poses a threat to personal health and its severity. If an individual feels that their health is threatened, a secondary assessment process is conducted to determine whether measures can be taken to deal with it [27,29]. This study believes that employees form occupational health risk perception in the primary assessment. In the process of secondary assessment, when employees think that the job demands exceed their perceived resources and endanger their health and happiness, work stress will occur [27]. The stronger the perception of occupational health risks, the greater the work stress.

The conservation of resources theory can also explain that the perception of occupational health risks will increase the work stress of employees. According to the resource conservation theory, employees like to preserve resources. When the resources they like (such as mental health and physical health) are at risk of loss or have been lost, pressure will arise [30]. Thus, we arrive at the following hypothesis:

**Hypothesis** **3a** **(H3a).** *Occupational health risk perception positively affects work stress.*

Organizational commitment refers to employees’ commitment to the organization, that is, the relative strength of employees’ identification and participation in a specific organization [31]. Organizational commitment includes three components, namely affective commitment, continuous commitment and normative commitment [32]. Affective commitment refers to employees’ emotional attachment, identification, and participation in the organization. Continuous commitment refers to employees’ evaluation of the relevant costs of leaving the organization. Normative commitment refers to employees’ commitment that they have an obligation to stay in the organization, which is an obligation to continue employment. This study holds that organizational commitment is a psychological state existing between employees and organizations, which can reduce the possibility of active resignation, and represents employees’ participation, loyalty, and identification with the organization [33].

Employees’ perceived characteristics of work and organization are generally considered to be the antecedents of organizational commitment [34,35]. Kivimaki and Kalimo (1993) [36] studied nuclear power plant workers and found that employees who estimated that accidents were more likely to happen had less organizational commitment. This study believes that employees with greater perception of occupational health risks tend to have less commitment to the organization. Hence, we posit the following hypothesis:

**Hypothesis** **3b** **(H3b).** *Occupational health risk perception negatively affects organizational commitment.*

### 2.6. The Impact of Work Stress and Organizational Commitment on Job Satisfaction

It has been confirmed that employees’ work stress negatively affects job satisfaction [37]. For example, Pignata et al. (2016) [38] found that employees who did not receive stress reduction intervention had significantly lower job satisfaction than employees who received stress reduction intervention. Work stress significantly affected nurses’ job satisfaction. Nurses with greater work stress felt lower job satisfaction [39,40]. O’Neill and Davis (2011) [5] took hotel employees as the research object, and found that work stress reduced hotel employees’ job satisfaction and had a harmful impact on employees’ productivity and work results. Therefore, this study puts forward the following hypothesis:

**Hypothesis** **4a** **(H4a).** *Work stress negatively affects job satisfaction.*

Numerous studies have shown that organizational commitment can positively promote job satisfaction. For example, Peng et al. (2016) [6] found through empirical research that job satisfaction and organizational commitment play an intermediary role between core self-evaluations and job burnout. At the same time, it is also confirmed that organizational commitment can positively promote job satisfaction. Organizational commitment is regarded as an important antecedent variable of job satisfaction. Employees with low organizational commitment are less likely to identify with and participate in the organization. Therefore, they get less reward from the organization, which leads to dissatisfaction with their work. With high organizational commitment, employees will be more actively involved in the work of the organization. Therefore, they will get more returns from the organization, which will further improve job satisfaction. Thus, this study puts forward the following hypothesis:

**Hypothesis** **4b** **(H4b).** *Organizational commitment positively affects job satisfaction.*

### 2.7. The Mediating Role of Work Stress and Organizational Commitment

The stress framework proposes that employees’ exposure to risk characteristics will lead to employees’ perceived stress [2]. Employees’ perception of occupational health risks will increase their stress. Work stress will further lead to employees’ mental health problems, physical health problems, and low job satisfaction [5]. Work stress plays a mediating role between occupational health risk perception and job satisfaction. Therefore, we hypothesize the following:

**Hypothesis** **5a** **(H5a).** *Occupational health risk perception negatively affects job satisfaction through work stress.*

Occupational health risk perception, as a negative job perception feature, will negatively affect employees’ commitment to the organization. Organizational commitment is regarded as an important antecedent variable of job satisfaction. Organizational commitment will further positively affect employees’ job satisfaction [6]. Organizational commitment plays a mediating role between occupational health risk perception and job satisfaction. Therefore, we hypothesize the following:

**Hypothesis** **5b** **(H5b).** *Occupational health risk perception negatively affects job satisfaction through organizational commitment.*

**Figure 1:**
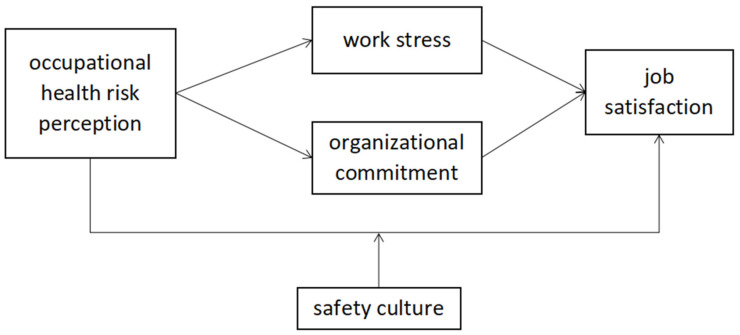
Research model.

To sum up, the research model constructed in this study is shown in Figure 1.

## 3. Methodology

### 3.1. Data Collection and Sample

This study used a questionnaire survey to verify the hypotheses. The informed consent was shown on the first page of the online questionnaire. All subjects gave their informed consent for inclusion before they participated in the study. As the questionnaire was anonymous, we told the respondents what the purpose of the study was during the survey and they helped us fill in the questionnaires. The questionnaires are from self-reports of employees. We did not collect any data without consent. Hence, the study was well approbated by them.

To ensure the reliability of the sample, we adopted a four-stage questionnaire development process. First, we interviewed five employees working in different enterprises in order to get a preliminary understanding of the specific situation of occupational health risks and job satisfaction. This provided support for the design of a formal questionnaire in this study. Second, we collated scales that had been cited internationally in relevant fields. On the basis of existing research, we designed a preliminary questionnaire combined with the interview information of employees. Thirdly, we conducted a small-scale pre-survey [41]. This study selected about 30 employees from several enterprises for a trial survey. We adjusted the questionnaire according to the trial investigation. Fourth, we summarized the important information in the above steps to improve the questionnaire and formed a formal questionnaire.

We started issuing questionnaires in October 2021. To improve the representativeness of the sample, team members collected questionnaires in several regions, including Shandong Province, Jilin Province, and Chongqing Municipality. These areas include both coastal developed areas and less developed inland areas. Due to the influence of COVID-19, the survey was conducted online, and questionnaires were distributed using mobile Internet social media and other emerging technologies. The research objects were employees of state-owned enterprises and private enterprises, including technical engineers (e.g., production line employees) and managers (e.g., grass-roots managers and middle-level managers). We collected a wide range of questionnaires from employees of state-owned enterprises and private enterprises because employees of different types of enterprises are exposed to occupational health risks. We randomly sent out about 500 questionnaires and eventually collected about 250 questionnaires.

We then screened these questionnaires. First, we checked the sample and eliminated questionnaires that clearly did not involve occupational health safety risks. Second, we eliminated the samples for which the completion rate did not reach 75%. At the same time, we also checked the key information of the questionnaire and excluded some enterprise samples whose key information was missing, such as employees’ working hours and job satisfaction. In the end, there were 237 valid samples and the characteristics of these samples are shown in Table 1.

### 3.2. Measures

In this study, a Likert seven-point scoring method was used for the scale measurement. All scales referred to the measurement methods that have been applied internationally. The specific measurements are as follows. 

Dependent variable: job satisfaction. The measurement of job satisfaction mainly referred to the study of Janssen (2001) [42] and adopted five questions to measure. For example, “I am satisfied with my job performance” and “I am satisfied with the way I work” (Cronbach’α = 0.914).

Independent variable: occupational health risk perception. Occupational health risk perception used seven items, mainly from the study of Liu et al. (2021) [1]. For instance, “frequent exposure to dust at work can lead to respiratory diseases (such as pneumoconiosis)” and “inhaling formaldehyde, benzene and other irritant gas will cause physical discomfort (such as headache)” (Cronbach’α = 0.885).

Mediating variables: work stress and organizational commitment. Firstly, according to Parker and Decotiis (1983) [43], we used 10 items to measure work stress. For example, “my work often prevents me from spending enough time with my family”, “my work leaves me short of leisure time”, and “work sometimes causes tightness in my chest” (Cronbach’α = 0.937). Secondly, as for the measurement of organizational commitment, we mainly referred to the study of Meyer, Allen, and Smith (1993) [44], which was divided into three dimensions: affective organizational commitment, sustainable organizational commitment, and normative organizational commitment. The measurement of organizational commitment consisted of nine items. For example, “I am happy to spend the rest of my career with this company”, “the company means a lot to me personally”, “there are so many things in my life that would be disrupted if I decided to leave my company”, and “even if it were in my interest, I don’t think it would be right to leave my company now” (Cronbach’α = 0.946).

Moderating variable: safety culture. We used the measurement of Cox and Cheyne (2000) [25] for reference and used nine items for measurement. For instance, “when there is a security problem, the management takes decisive action”, “management clearly believes that employee safety is important”, “I am strongly encouraged to report unsafe situations”, and “It is a safer place to work than other companies I have worked for” (Cronbach’α = 0.965).

Control variables. We set several control variables, including work experience, educational background, and enterprise size. First, work experience reflected how long an employee had worked in an enterprise. Second, educational background meant the education of employees (4 = Master’s degree or PhD; 3 = bachelor’s degree; 2 = junior college; 1 = high school or below). Thirdly, the size of an enterprise reflected the number of employees it has (4: more than 200; 3: 51–200, 2: 21–50, 1: 20 or less).

### 3.3. Common Method Bias

The Harman’s one-factor test was conducted, which was widely used among empirical studies. From the result, we could see that the largest factor only explained 29.781% of the entire variance. Based on the view of Podsakoff and Organ (1986) [45], it shows that this study did not appear to have the problem of common method bias.

## 4. Data Analysis

The descriptive statistics and correlations among main variables are shown in Table 2. The results show that the mean and standard deviation of each variable has no singular value. The correlation coefficients between the core variables did not exceed 0.6. Subsequently, we tested the multicollinearity issue. The values of variance inflation factors (VIFs) did not exceed 10. According to Hair et al. (1998) [46], it showed that there was no significant multicollinearity. 

Hierarchical linear analysis was utilized to test the hypotheses of this study. We built several models, and the results are shown in Table 3. In model 1, we verified the influence of each control variable on the dependent variable’s job satisfaction. Model 2 was used to verify H1. The results show that the coefficient for occupational health risk perception was −0.134 (Model 2: β = −0.134; *p* < 0.05). This shows that occupational health risk perception negatively influenced job satisfaction. Therefore, hypothesis 1 is supported by the samples. In order to verify hypothesis H2, we constructed model 3. The results of model 3 show that the interaction (safety culture × occupational health risk perception) was not significant (Model 3: β = −0.018; ns). The results show that assuming H2, the moderating effect of safety culture was not supported by the data.

Subsequently, we constructed model 4 and model 5 to verify H3a and H3b. The coefficient for occupational health risk perception was 0.150 (Model 4: β = 0.150; *p* < 0.05). The results suggest that occupational health risk perception positively affected work stress. At the same time, the coefficient for occupational health risk perception was −0.149 (model 5: β = −0.149; *p* < 0.05). The results indicated that occupational health risk perception negatively affected organizational commitment. Therefore, both H3a and H3b were supported by the data.

Model 6 was used to verify H4a and H4b (see Table 4). The coefficient for work stress was −0.169 (Model 6: β = −0.169; *p* < 0.05). The coefficient for organizational commitment was 0.415 (Model 6: β = 0.415; *p* < 0.001). Therefore, the data supported hypothesis H4a, that work stress negatively affects job satisfaction, and hypothesis H4b, that organizational commitment positively affects job satisfaction.

In order to verify the mediating effect of work stress, we added the mediating variable work stress to model 2 and constructed model 7. The results of model 7 show that the significance of occupational health risk perception decreased after the inclusion of mediating variables (see Table 4). Combined with the results of model 2, model 6, and model 7, the data supported hypothesis H5a, that occupational health risks negatively affect job satisfaction through work stress. In model 8, organizational commitment variables were added to model 2 to verify hypothesis H5b. Similarly, combined with the results of model 2, model 6, and model 8, hypothesis H5b, namely occupational health risks negatively affect job satisfaction through organizational commitment, was supported by the data.

## 5. Discussion

This study aims to explore the influence mechanism of occupational health risk perception on employees’ job satisfaction. Taking enterprise employees as the research object, we collected information such as employees’ perception of occupational health risks, work stress, organizational commitment, and job satisfaction through the questionnaire survey, and conducted hypothesis testing through empirical methods. According to the results of empirical analysis, we found that employees’ perception of occupational health risks will significantly and negatively affect employees’ job satisfaction. Work stress and organizational commitment play a mediating role between occupational health risk perception and job satisfaction.

First, employees’ perception of occupational health risks will significantly and negatively affect employees’ job satisfaction. Therefore, enterprise managers should attach great importance to the safety characteristics of the workplace, reduce employees’ perception of occupational health risks, support employees voice, and improve employees’ job satisfaction [47], so as to achieve the goal of building a harmonious organization. This conclusion is consistent with the findings of Thoresen et al. (2003) [4], that is, occupational health risk perception, as a negative job perception feature, will negatively affect employees’ job satisfaction.

Second, work stress plays a mediating role between occupational health risk perception and job satisfaction. In the workplace, the less the employees’ perception of occupational health risks, the lower their work stress, and they will then form higher job satisfaction. This conclusion further confirms the research of Meurs and Perrewe (2011) [27] and Wu et al. (2019) [37]. In the workplace, employees will assess work environment events. Employees form occupational health risk perception in the primary assessment and the secondary assessment process will form work stress. Work stress, as a negative psychological state, will reduce employees’ job satisfaction.

Thirdly, organizational commitment plays a mediating role between occupational health risk perception and job satisfaction. In the workplace, the less employees’ perception of occupational health risks, the higher their commitment to the organization, and they will form higher job satisfaction. This conclusion further confirms the research of Kivimaki and Kalimo (1993) [36] and Peng et al. (2016) [6]. Through the research, they found that employees with greater awareness of occupational health risks have lower organizational commitment. Employees with low organizational commitment have lower recognition and participation in the organization, so they get less return from the organization, which will lead to dissatisfaction with their work.

Fourth, in this study, the data we collected cannot support hypothesis H2, that safety culture weakens the negative impact of occupational health risk perception on job satisfaction. According to the job demand-resources model, Nielsen et al. (2011) [12] took safety critical organization as the research object and confirmed the moderating effect of safety culture on risk perception and job satisfaction. There may be several reasons why our study failed to verify the moderating effect of safety culture. This may be because the sample size we collected was small and the sample was not rich enough. In the future, we need to expand the sample size and use more samples to test this hypothesis. This may also reflect that the current enterprises do not pay enough attention to the construction of safety culture or take safety into consideration, which makes it difficult for employees to perceive safety culture at work, and makes it difficult for us to find the value of safety culture in actual research. This phenomenon should be paid attention by enterprises.

Theoretical contributions. The results of this study have a certain theoretical contribution. Firstly, this study integrates the relevant knowledge of occupational health and risk perception, pays attention to the emerging concept of occupational health risk perception, and summarizes the literature of occupational health risk perception. This will help scholars to further explore and study the perception of occupational health risks. We found that occupational health risk perception of employees has a great impact on employees’ work attitude and work outputs, which needs to be paid more attention by scholars and practitioners [48]. Organizations and managers need to take steps to reduce the impact of occupational health risks on employees. Second, this study enriches the research on the influence mechanism of job perception characteristics on job satisfaction. Previous studies have mainly focused on the impact of occupational health risk perception on safety behavior and work behavior, and lack research on the impact mechanism between occupational health risk perception and job satisfaction. In fact, employees’ job satisfaction has an extremely important impact on organizational construction. Occupational health risk perception is an important factor influencing job satisfaction, and the study on the influencing mechanism between occupational health risk perception and job satisfaction has certain theoretical significance. Therefore, this study reveals the impact mechanism of occupational health risk perception on job satisfaction, and enriches the research on the impact mechanism of job perception characteristics on job satisfaction. Third, this study finds that employees’ work stress and organizational commitment are two paths for occupational health risk perception to affect job satisfaction. This discovery not only opens the black box of occupational health risk perception and job satisfaction, but also enriches the research on work stress and organizational commitment.

Practical contributions. The conclusion of this study also has a certain practical contribution. For enterprise managers, this study helps managers more deeply understand the importance of workplace safety characteristics and realize that occupational health risk perception will not only directly affect job satisfaction, but also indirectly affect job satisfaction through work stress and organizational commitment. Enterprise managers should plan to improve the safety characteristics of the workplace, reduce employees’ occupational health risk perception, and improve job satisfaction, so as to build a harmonious organization. For enterprise employees, this study helps them to understand the influencing factors of job satisfaction. Sometimes they may not have a particularly strong perception of occupational health risks, but when they have high work stress, low organizational commitment, or low job satisfaction, it is necessary for them to consider whether there are unsafe and unhealthy factors in their workplace. This will encourage employees to pay attention to their subjective risk perception, which will affect their psychological state, and then affect their mental health and physical health. This will also encourage employees the actively participate in the safety management of the enterprise, conduct creative behaviors [49], and work with enterprise managers to build a safe and healthy workplace. For organizations, reduced job satisfaction can have huge consequences and costs [12]. It is necessary for organizations to take intervention measures to reduce the impact of perceived occupational health risks on employees’ job satisfaction. The organization can regularly check the work stress of employees, and adopt lectures, games, and other methods to adjust the work stress of employees. Organizations should give more consideration to the internal needs of employees to support their sense of belonging to the organization and improve their organizational commitment [50]. In addition, organizations can improve employee job satisfaction by adopting good health programs [18].

Limitations and future research. This study has the following limitations, which we hope can be strengthened in future research. First, this study takes enterprise employees as the research object, focuses on the emotional part of occupational health risk perception, and pays insufficient attention to the rational part of occupational health risk perception. Future research should comprehensively consider the rational part and perceptual part of occupational health risk perception; collect data from employees, managers, and experts; and measure occupational health risk perception more comprehensively. Second, this study does not fully consider the impact of industry factors, and the sample size is limited. We encourage future research to collect data from multiple industries, expand the sample size, and enhance the persuasiveness of the research. Future research can continue to explore whether there are significant differences in research results among different industries. Third, this study was carried out at the individual level, focusing on employees’ subjective perception and evaluation. Future research can be extended to the team or organization level to further focus on whether occupational health risk perception will have an impact on team cooperation and organizational performance.

## 6. Conclusions

In conclusion, this study helps to better understand the relationship between occupational health risk perception, work stress, organizational commitment, and job satisfaction. This study also reveals the influence mechanism of occupational health risk perception on job satisfaction. Based on the empirical test, we find that the perception of occupational health risks will significantly and negatively affect employees’ job satisfaction. Work stress and organizational commitment play a mediating role between occupational health risk perception and job satisfaction. This study not only helps enterprise managers realize that reducing occupational health risk perception will improve employees’ job satisfaction, but also encourages employees to actively participate in workplace safety improvement and actively improve job satisfaction. Therefore, this study has certain theoretical and practical significance.

## Figures and Tables

**Table 1:** The profiles of the samples.

Characteristics		N	Percentage
Work age	3 years or less	48	20.25%
	3–10 years	71	29.96%
	More than 10 years	118	49.79%
Firm size (number of employees)	1–20	17	7.2%
	21–50	42	17.7%
	51–200	34	14.3%
	More than 200	144	60.8%
Founder’s education background	High school or below	18	7.6%
	Junior college	55	23.2%
	Bachelor degree	130	54.9%
	Master degree or Ph.D	34	14.3%

**Table 2:** Descriptive statistics and correlation matrix.

	Mean	S.D.	1	2	3	4	5	6	7	8
Work experience	5.80	4.214	1							
Education background	2.76	0.790	−0.074	1						
Firm size	3.83	1.420	0.186 *	0.359 ***	1					
Occupational health risk perception	4.370	1.752	0.149	−0.021	−0.079	1				
work stress	4.327	1.508	−0.046	0.041	0.034	0.139 *	1			
organizational commitment	4.466	1.468	0.043	−0.044	−0.014	−0.142 *	−0.106	1		
safety culture	5.431	1.251	0.095	−0.110	−0.021	0.058	−0.138 *	0.443 ***	1	
job satisfaction	5.185	1.206	0.087	−0.135 *	0.045	−0.130 *	−0.216 **	0.439 ***	0.547 ***	1

Note: * *p* < 0.05; ** *p* < 0.01; *** *p* < 0.001.

**Table 3:** The results of the regression analysis (model 1–5).

Variables	Job Satisfaction			Work Stress	Organizational Commitment
	Model 1	Model 2	Model 3	Model 4	Model 5
Control variables	Beta	Beta	Beta	Beta	Beta
Work experience	0.045	0.062	0.031	−0.055	0.048
Education background	−0.168 *	−0.165 *	−0.104	0.025	−0.038
Firm size	0.099	0.085	0.077	0.045	−0.019
Independent variables					
Occupational health risk perception		−0.134 *	−0.146 *	0.150 *	−0.149 *
Moderating variables					
safety culture			0.553 ***		
safety culture * Occupational health risk perception			−0.018		
*R* ^2^	0.030	0.148	0.338	0.159	0.157
Adj-*R*^2^	0.018	0.131	0.321	0.148	0.148
F-value	2.419	2.901 *	19.567 ***	11.496 ***	11.458 ***

Note: * p < 0.05; *** *p* < 0.001.

**Table 4:** Results of the regression analysis (model 6–8).

Variables	Dependent Variable: Job Satisfaction		
	Model 6	Model 7	Model 8
Work experience	0.027	0.052	0.042
Education background	−0.146 *	−0.160 *	−0.149 *
Firm size	0.105	0.093	0.092
Independent variables			
Occupational health risk perception		−0.104	−0.070
Mediating variables			
work stress	−0.169 **	−0.197 **	
organizational commitment	0.415 ***		0.422 ***
*R* ^2^	0.245	0.285	0.211
Adj-*R*^2^	0.229	0.266	0.205
F-value	14.987 ***	14.315 ***	13.144 ***

Note: * *p* < 0.05; ** *p* < 0.01; *** *p* < 0.001.

## Data Availability

The data presented in this study are available upon request from the corresponding author.
